# Supplementary figures and images for: Variation in Rural African Gut Microbiota Is Strongly Correlated with Colonization by Entamoeba and Subsistence
Source: PLoS Genet. 2015 Nov 30;11(11):e1005658. doi: 10.1371/journal.pgen.1005658 (PMC4664238; doi:10.1371/journal.pgen.1005658)

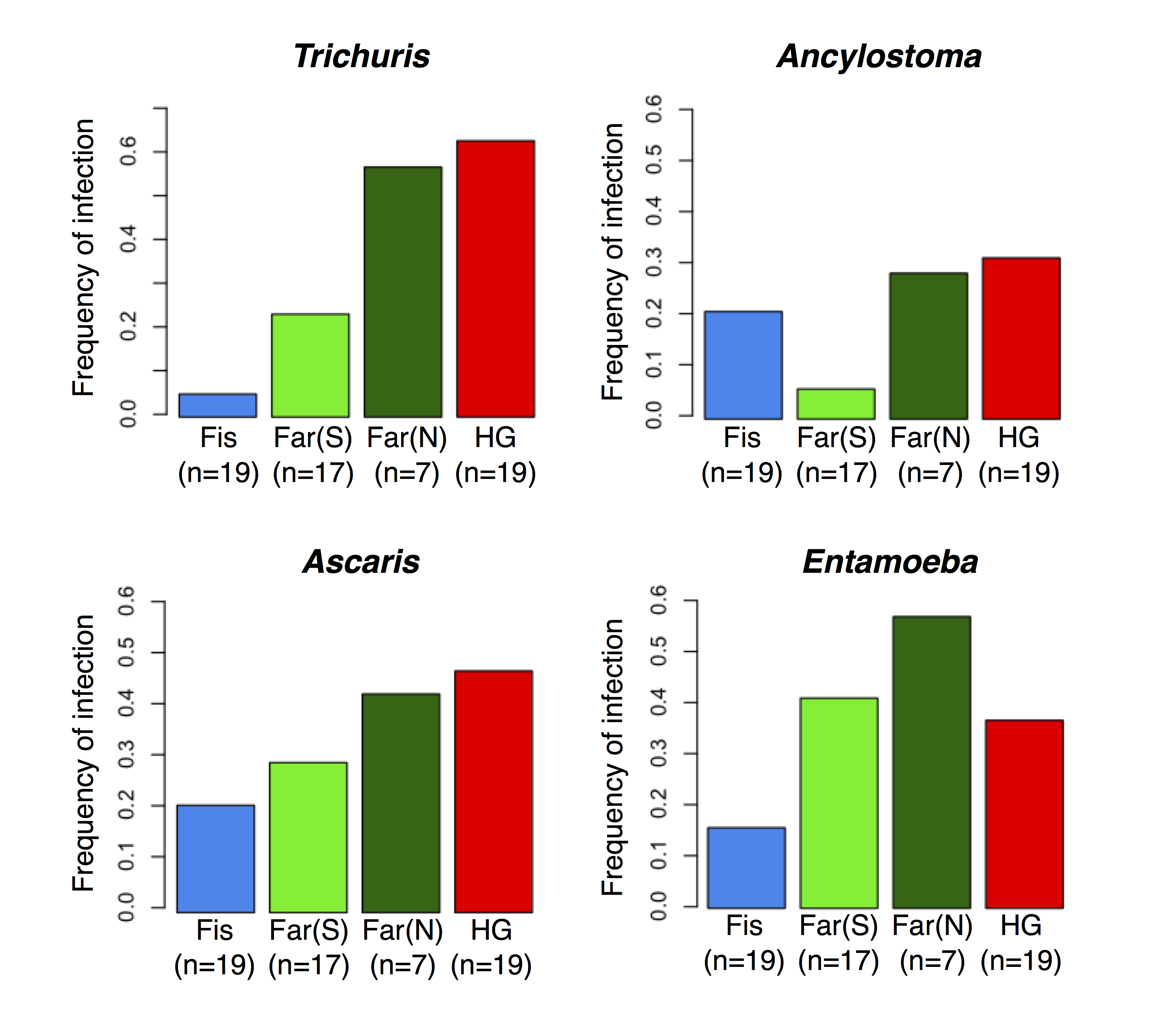

Supplement: S1 Fig — Prevalence of individual infection by four parasites (Trichuris, Ascaris, Ancylostoma, and Entamoeba) across the four subsistence groups. Fis = fishing population, Far(S) = farmers from the South, Far(N) = farmers from the North, and HG = hunter-gatherers. The total number of samples in each population (n) is indicated below the bar. Parasitism was assessed as either presence or absence of the species surveyed. (TIFF) [file pgen.1005658.s008.tiff]

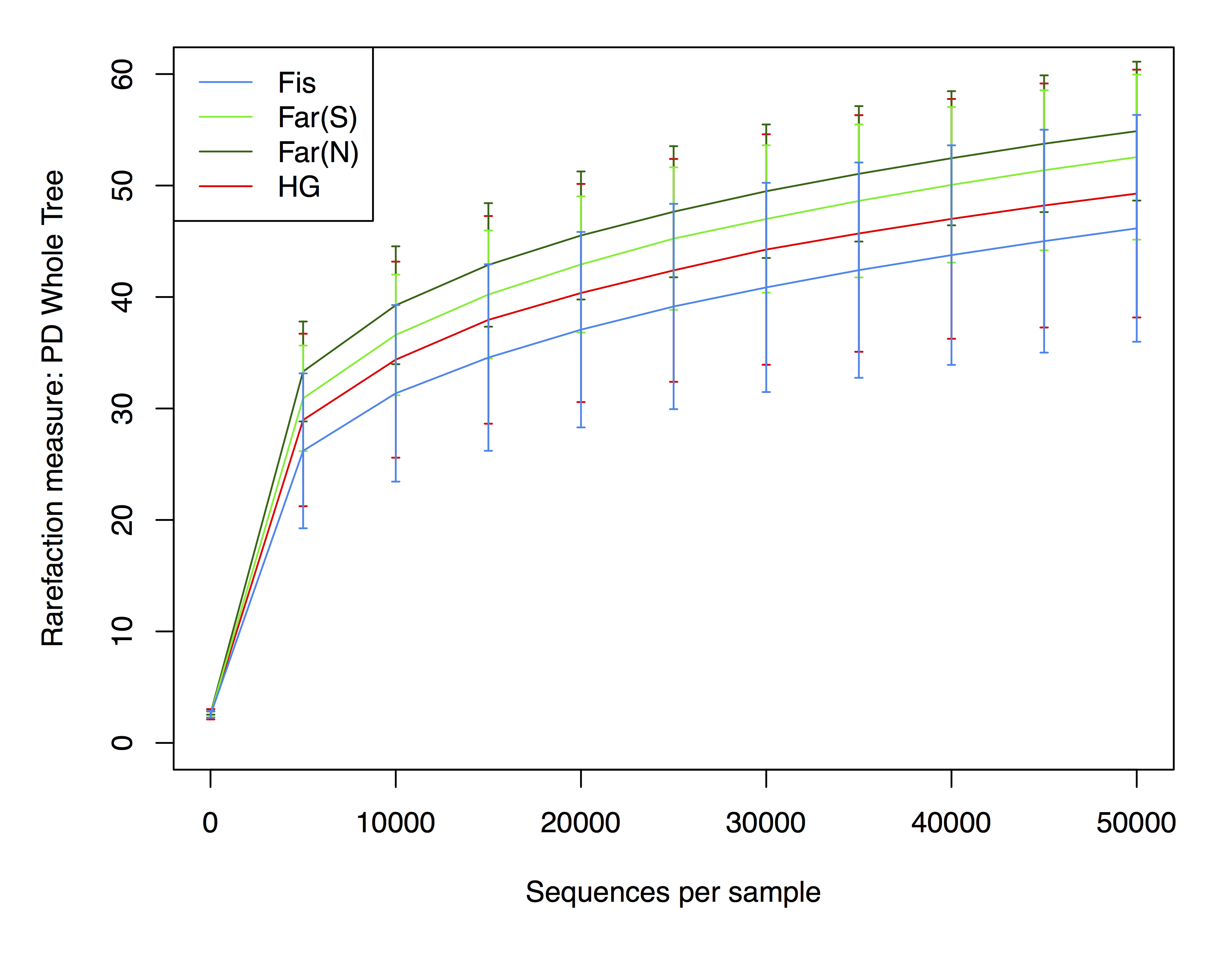

Supplement: S2 Fig — Alpha rarefaction curves for the four subsistence groups using the Phylogenetic Distance Whole Tree metric for alpha diversity (means across 10 iterations). Fis = fishing population, Far(S) = farmers from the South, Far(N) = farmers from the North, and HG = hunter-gatherers. Variance is among individuals. All further analyses are based on 50,000 reads per sample, the maximal depth with which we do not exclude any individuals. (TIFF) [file pgen.1005658.s009.tiff]

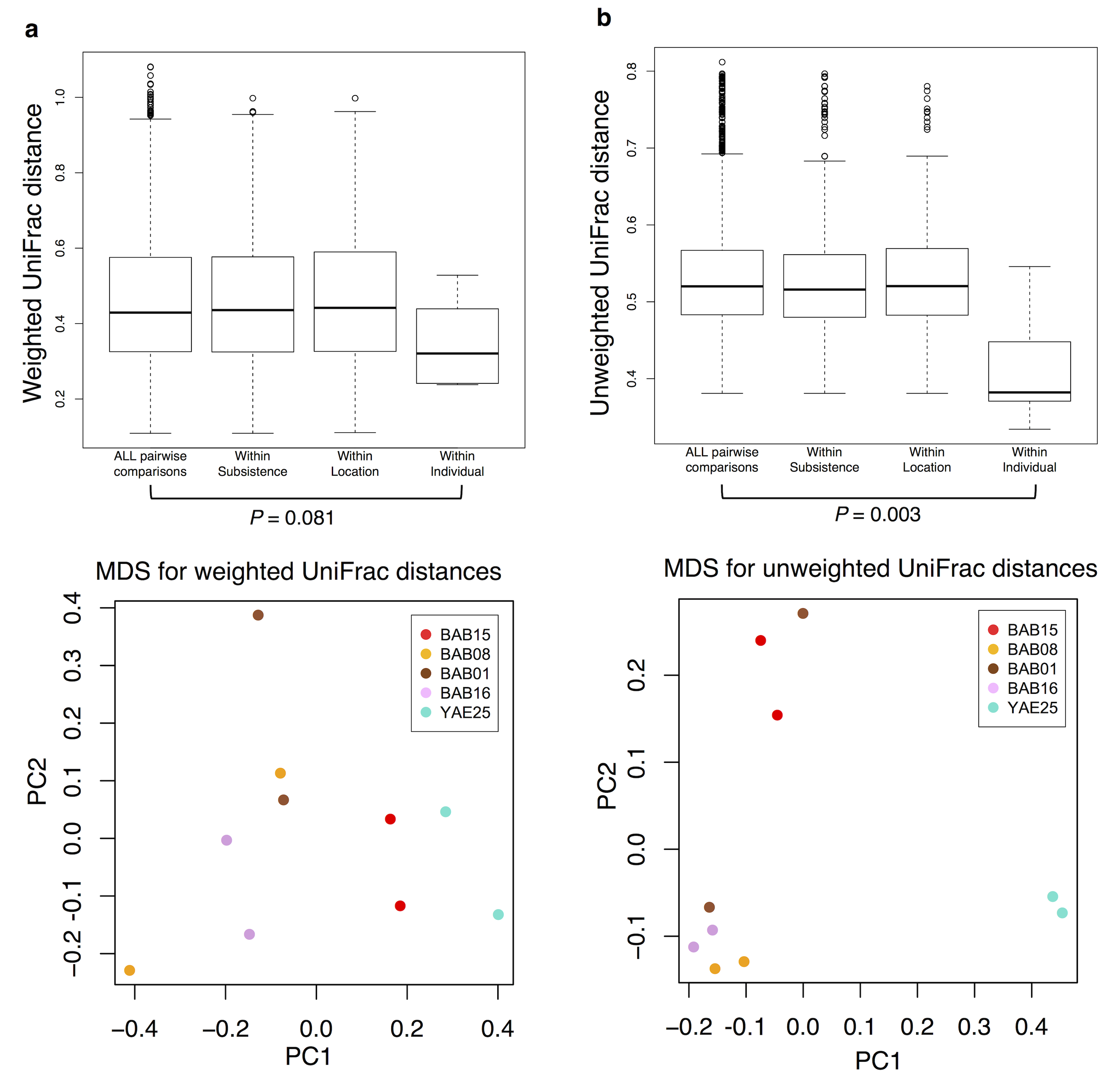

Supplement: S3 Fig — (a) Beta diversity estimates between all pairs of individuals, for pairs within subsistence groups, within locations, and within individuals based on weighted (left panel) and unweighted (right panel) UniFrac distances. P-values are from a Wilcox Rank Sum test. (b) Multidimensional Scaling (MDS) showing separation among replicate samples for 5 individuals using weighted (left panel) and unweighted (right panel) UniFrac distances. The first two principal components are shown on the axes. (TIFF) [file pgen.1005658.s010.tiff]

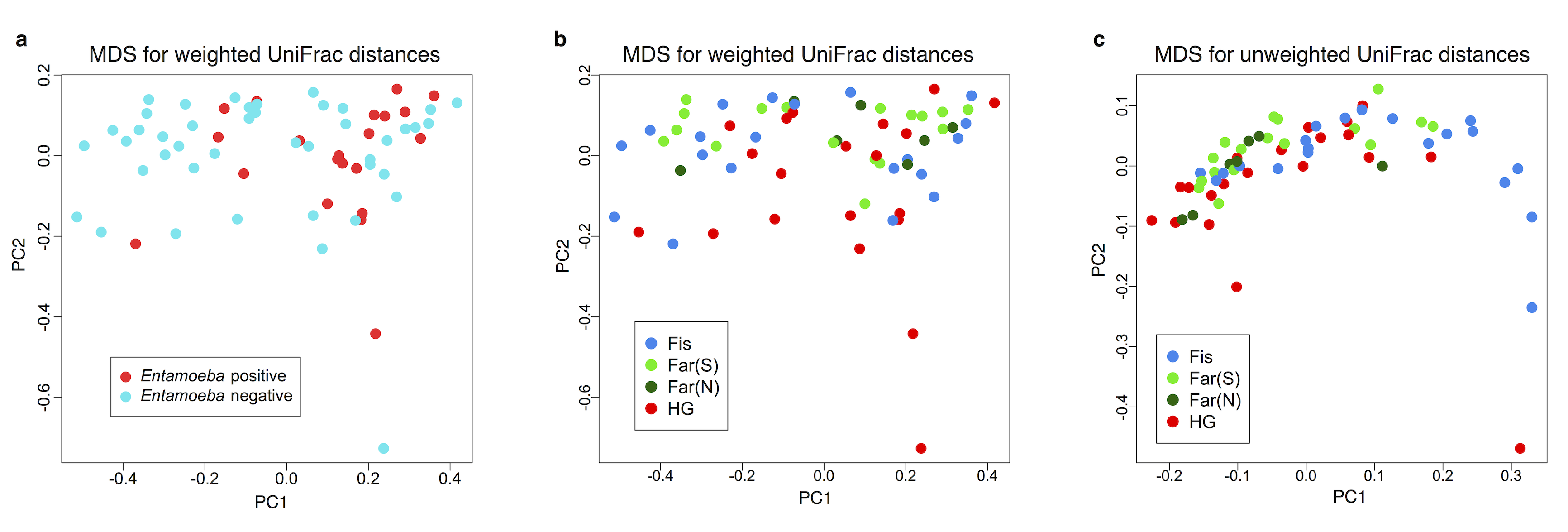

Supplement: S4 Fig — Multidimensional Scaling plots for (a) weighted UniFrac distances colored by Entamoeba status (b) weighted UniFrac distances colored by subsistence (Fis = fishing population, Far(S) = farmers from the South, Far(N) = farmers from the North, and HG = hunter-gatherers), and (c) unweighted UniFrac distances colored by subsistence. For each plot, the first two principal components (PC1 and PC2) are shown. (TIFF) [file pgen.1005658.s011.tiff]

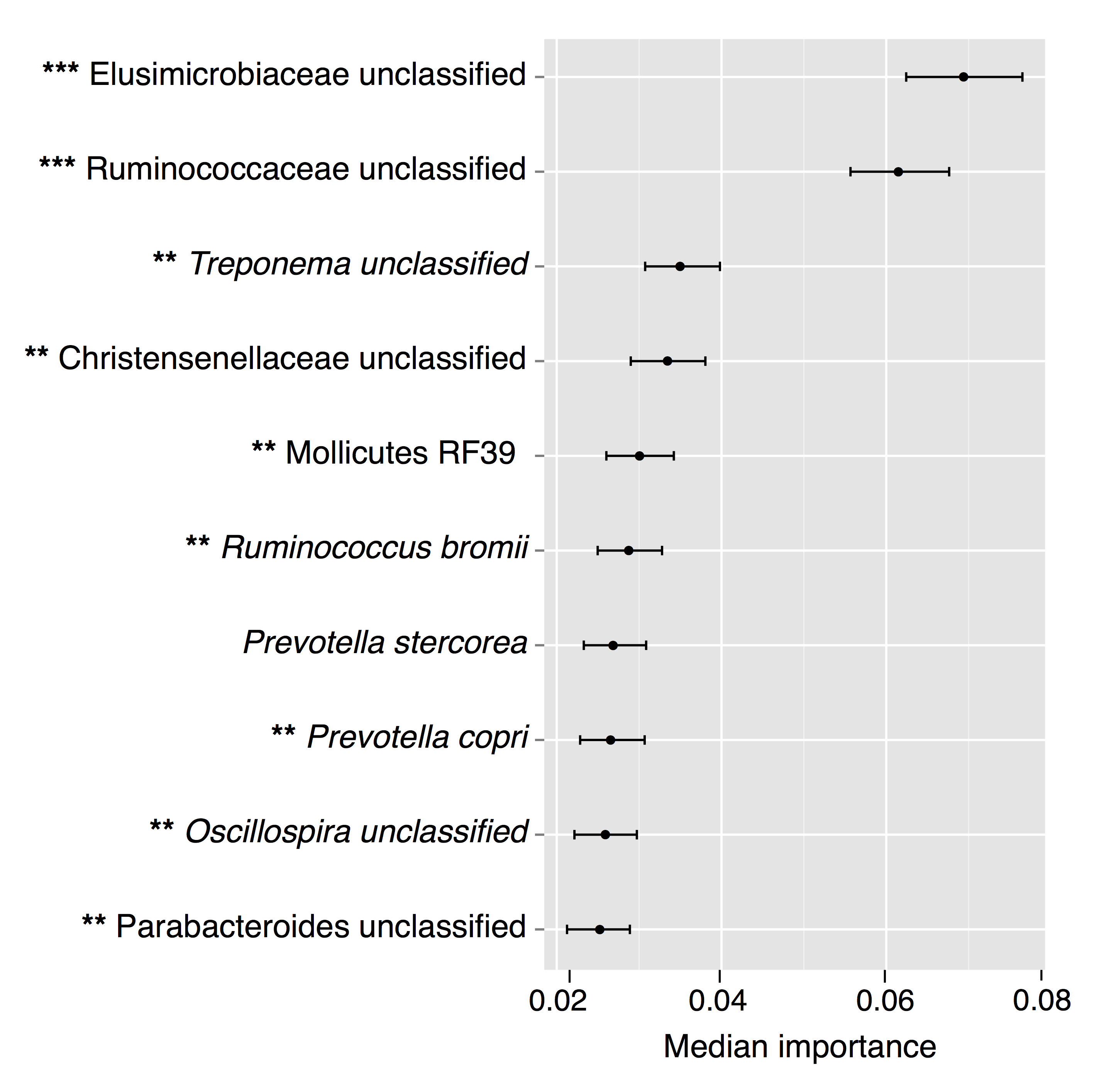

Supplement: S5 Fig — Summary of the ten most discriminating taxa identified by a random forest classifier (RFC) model to be predictive of Entamoeba infection status. A RFC with 2000 decision trees was trained on the data (relative abundances of all taxa occurring at > = 0.1% in at least 4 individuals) with 5-fold cross-validation. Importance values were calculated as the mean decrease in node impurity. 95% confidence intervals from 1000 random forests are shown. Mean accuracy over the 5 folds was 0.79 (SD = 0.09). P < 0.001, estimated using 1000 permutation tests. Taxa that were identified as being significant in an ANOVA are indicated by asterisk(s) (*); q < 0.0001 (***), q < 0.0001 (**), q < 0.001 (*). (TIFF) [file pgen.1005658.s012.tiff]

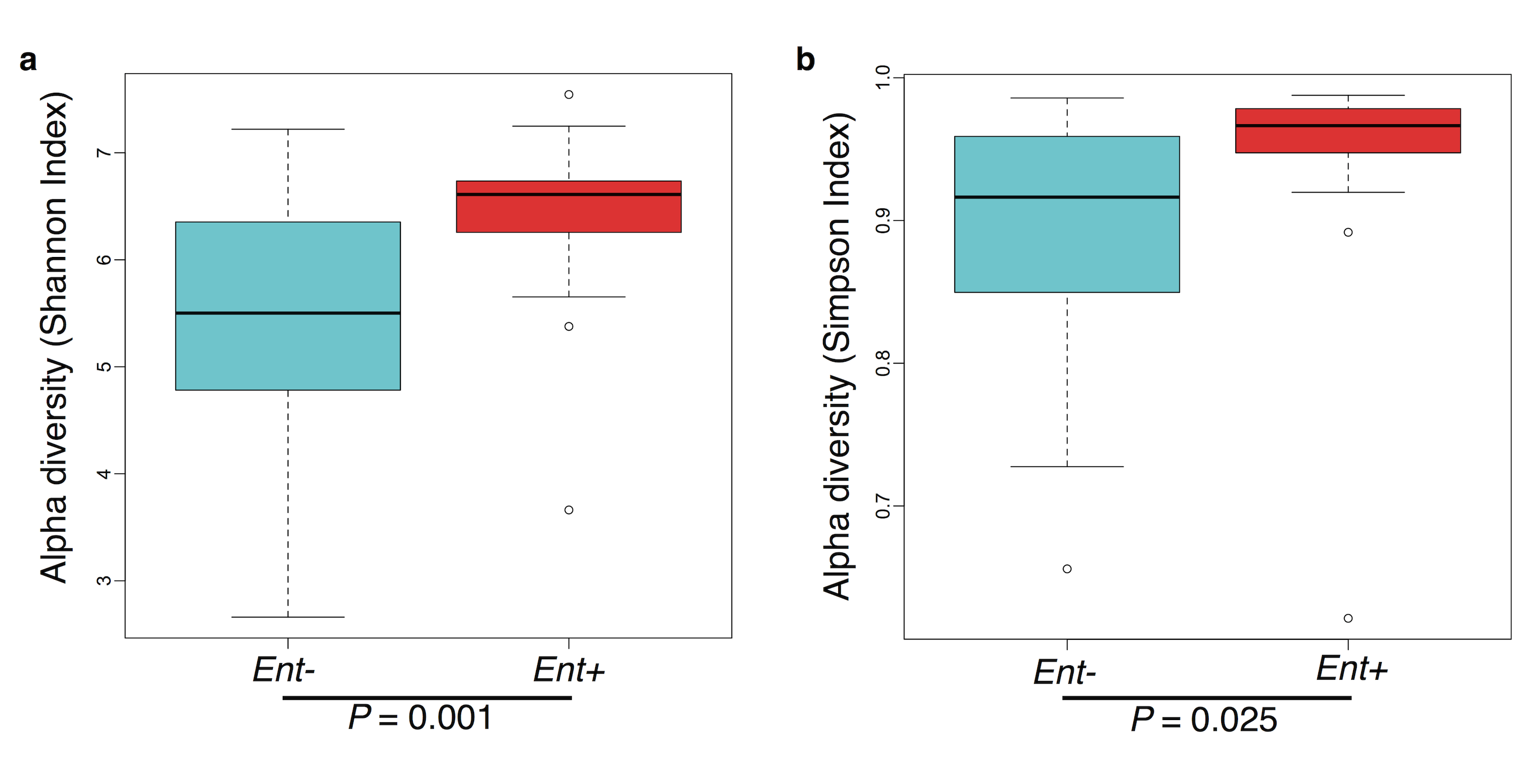

Supplement: S6 Fig — Comparison of alpha diversity for Entamoeba negative (Ent-) and positive (Ent+) individuals using the Shannon Index (a) and Simpson’s Index (b). P-values are based on a Welch’s t-test. (TIFF) [file pgen.1005658.s013.tiff]

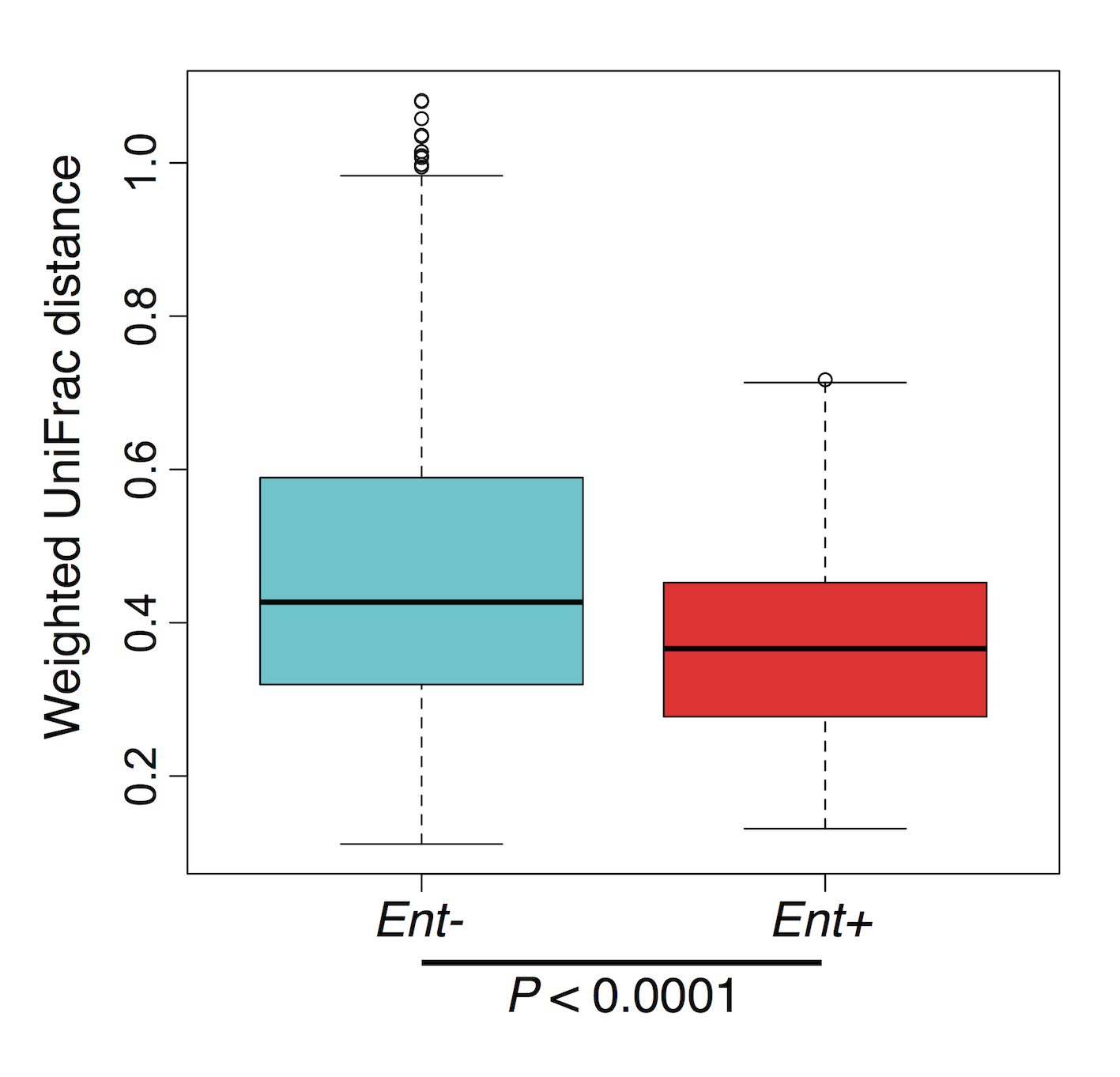

Supplement: S7 Fig — Comparison of beta diversity for Entamoeba negative (Ent-), positive (Ent+), and between Ent- and Ent+ individuals based on weighted UniFrac distances. P-values are based on a Welch’s t-test. (TIFF) [file pgen.1005658.s014.tiff]

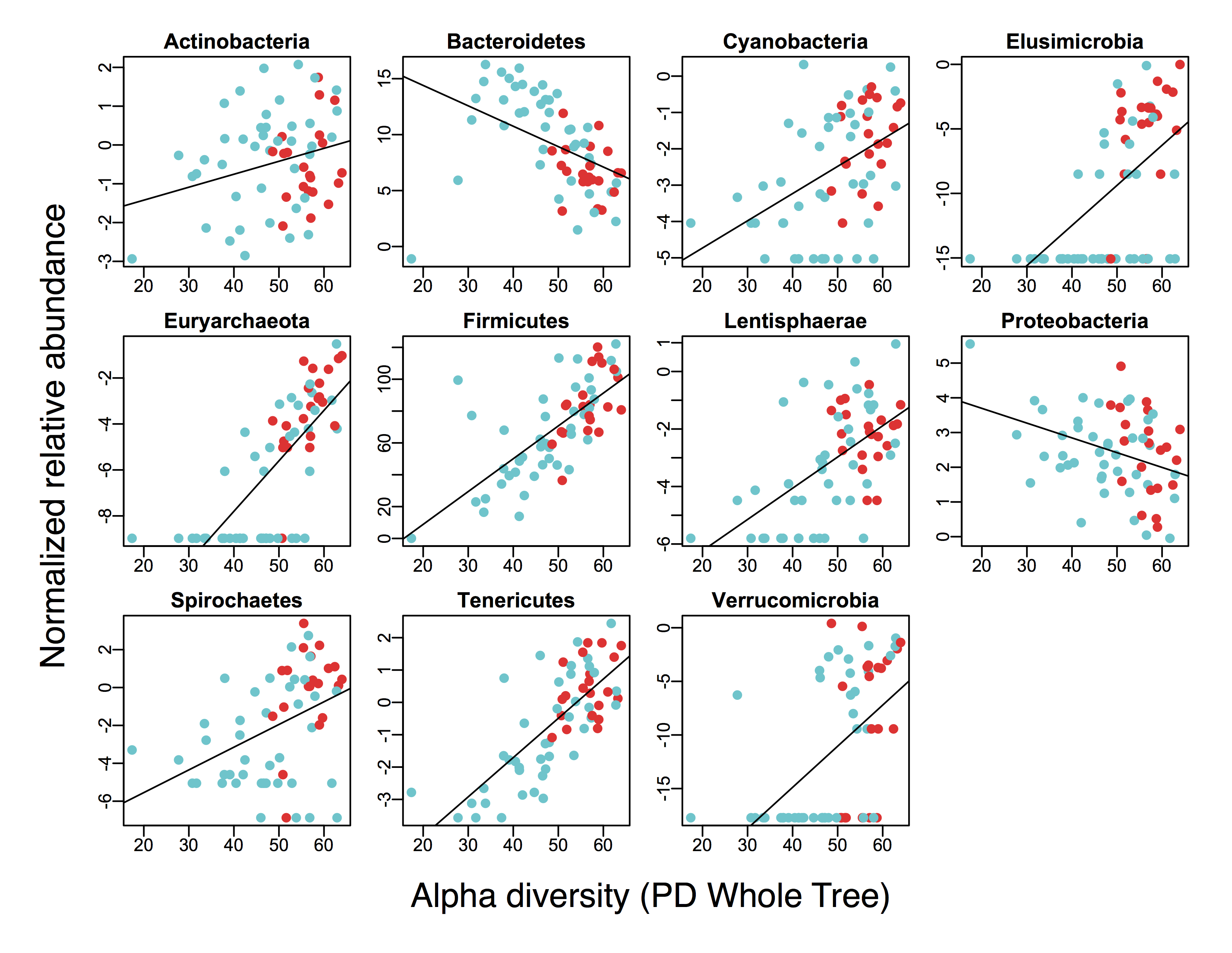

Supplement: S8 Fig — Phyla (occurring at > = 0.1% in at least 4 individuals) for which abundance is significantly correlated with alpha diversity (phylogenetic distance whole tree metric) in Entamoeba negative (Ent-, blue) and positive (Ent+, red) individuals. q < 0.05; ANOVA. (TIFF) [file pgen.1005658.s015.tiff]

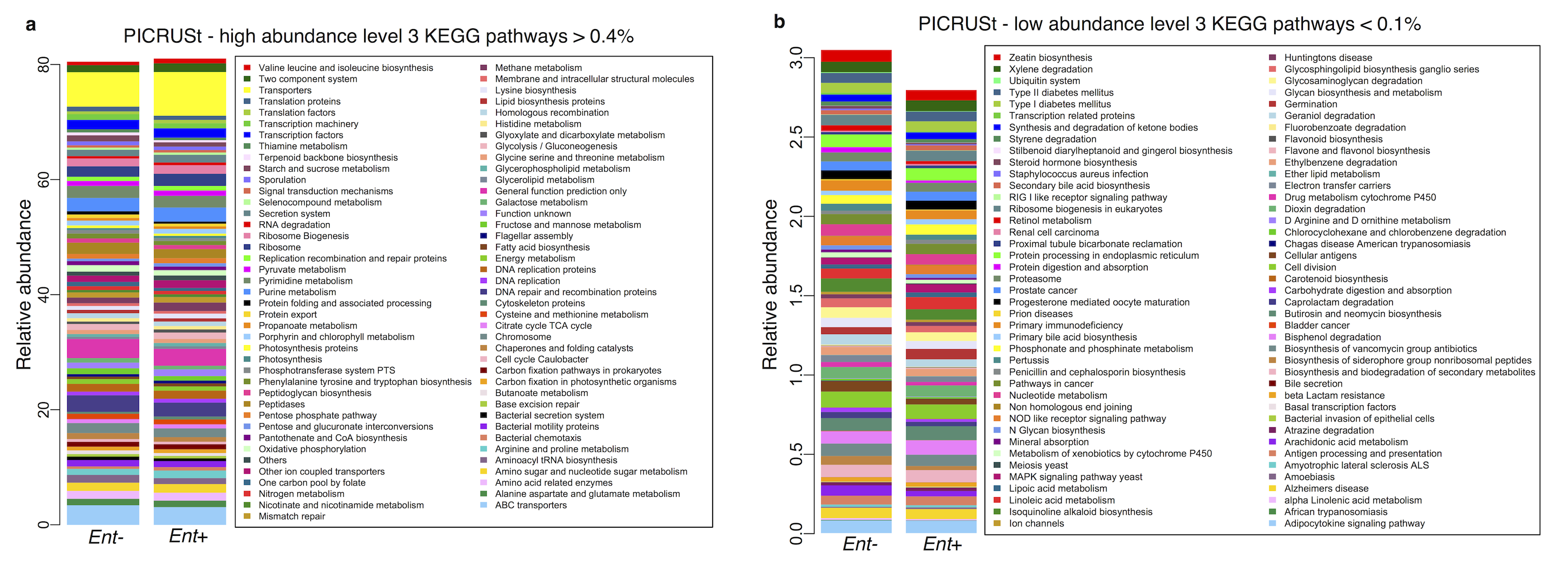

Supplement: S9 Fig — Summary of predicted metagenomic composition for Entamoeba negative (Ent-) and positive (Ent+) individuals based on phylotypic investigation of communities by reconstruction of unobserved states (PICRUSt). Relative abundance of (a) the most abundant (> = 0.4% in at least one group) and (b) least abundant (< = 0.1% in at least one group) of KEGG (Level 3) pathways. (TIFF) [file pgen.1005658.s016.tiff]

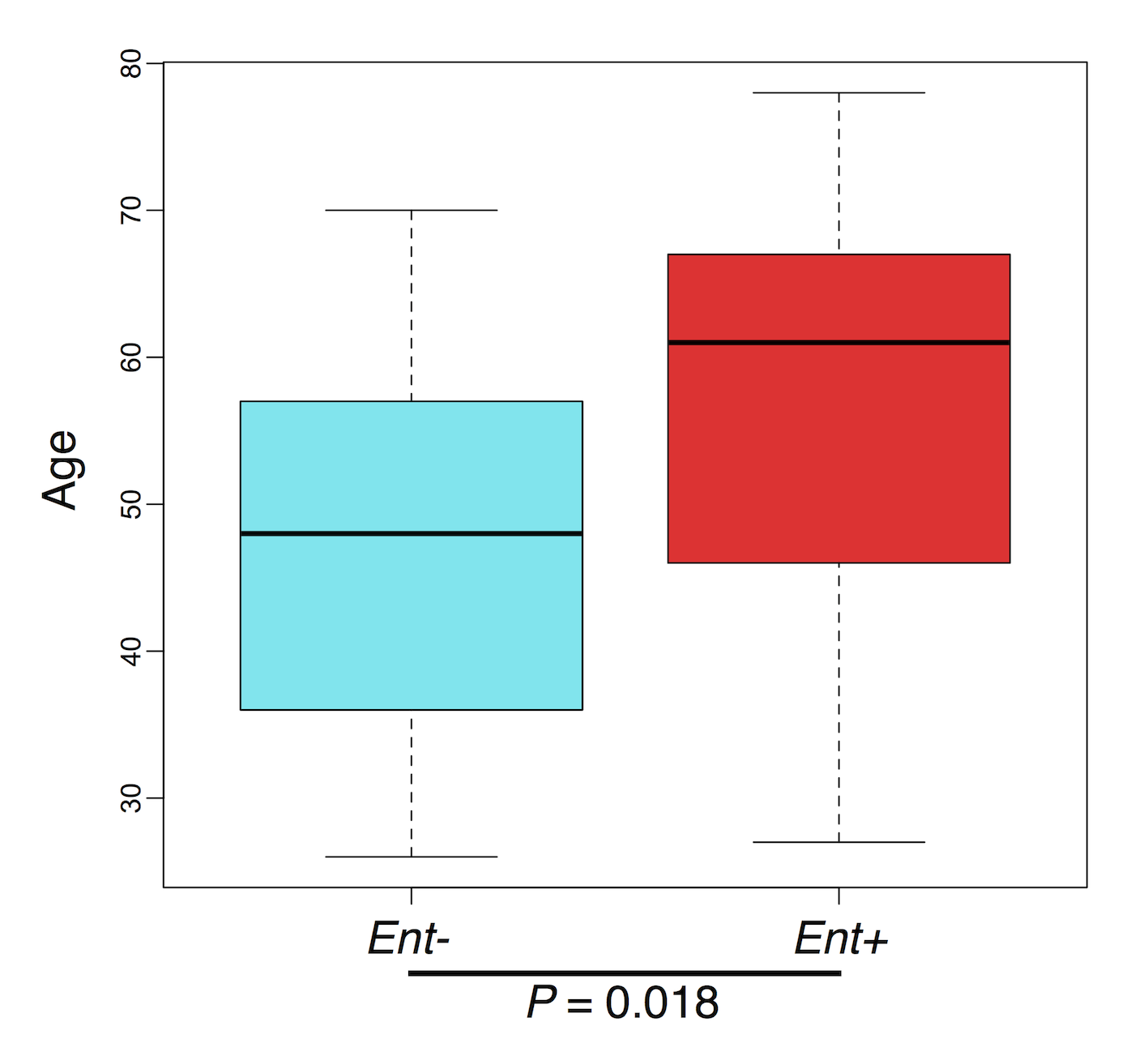

Supplement: S10 Fig — Mean age of Entamoeba negative (Ent-) and positive (Ent+) individuals. P-value are based on a Welch’s t-test. (TIFF) [file pgen.1005658.s017.tiff]

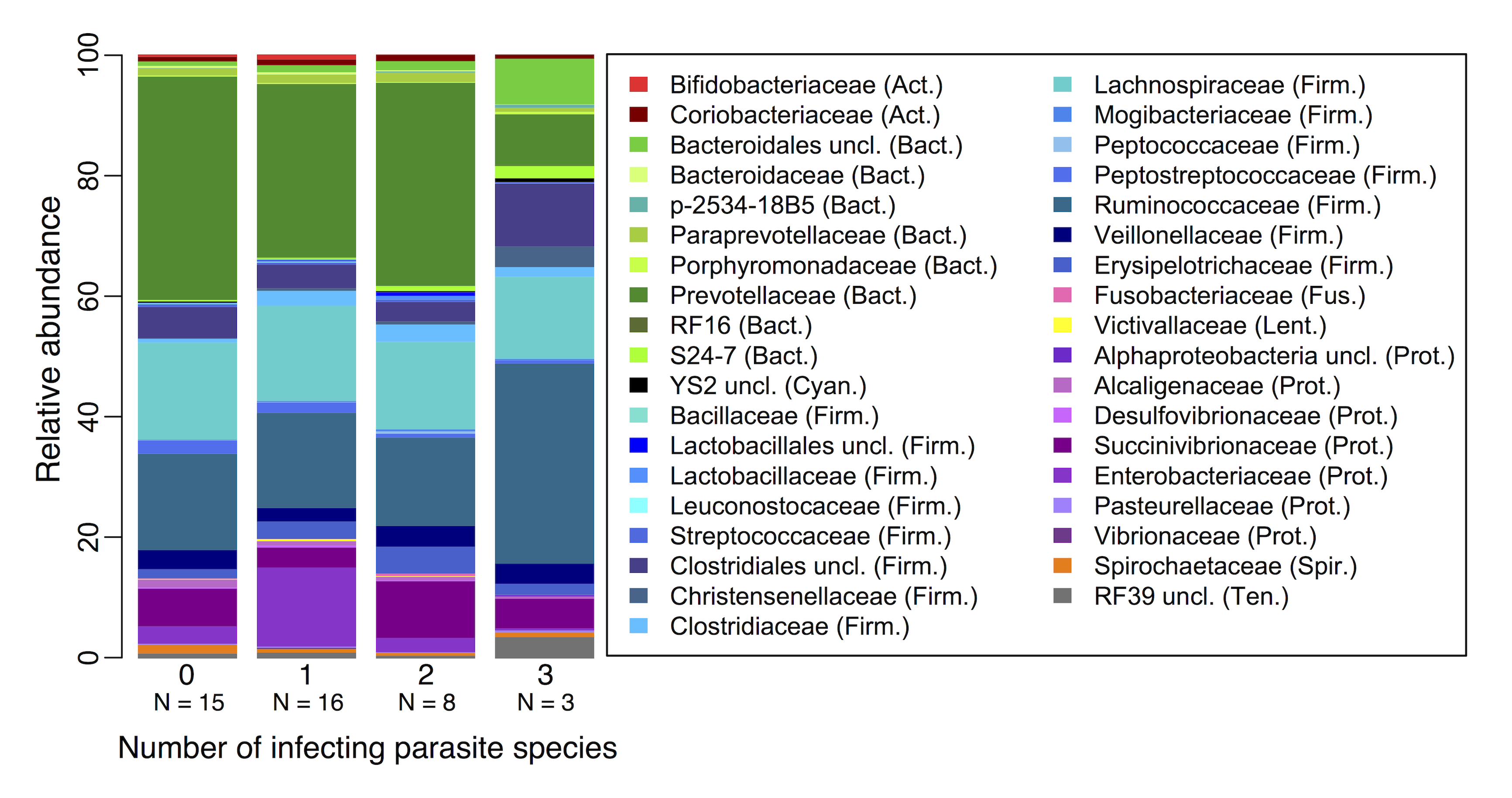

Supplement: S11 Fig — Summary of the relative abundance of taxa (> = 0.1% in at least 4 individuals) for the number of infecting parasites in the absence of Entamoeba infection (0–3). Taxa are colored by phylum (Acinobacteria (Act.) = red, Bacteroidetes (Bact.) = green, Cyanobacteria (Cyan.) = black, Elusimicrobia (Elus.) = gold, Firmicutes (Firm.) = blue, Fusobacteria (Fus.) = pink, Lentisphaerae (Lent.) = yellow, Proteobacteria (Prot.) = purple, Spirochaetes (Spir.) = orange, and Tenericutes (Ten.) = gray). The number of individuals (N) that falls into each category is indicated. (TIFF) [file pgen.1005658.s018.tiff]

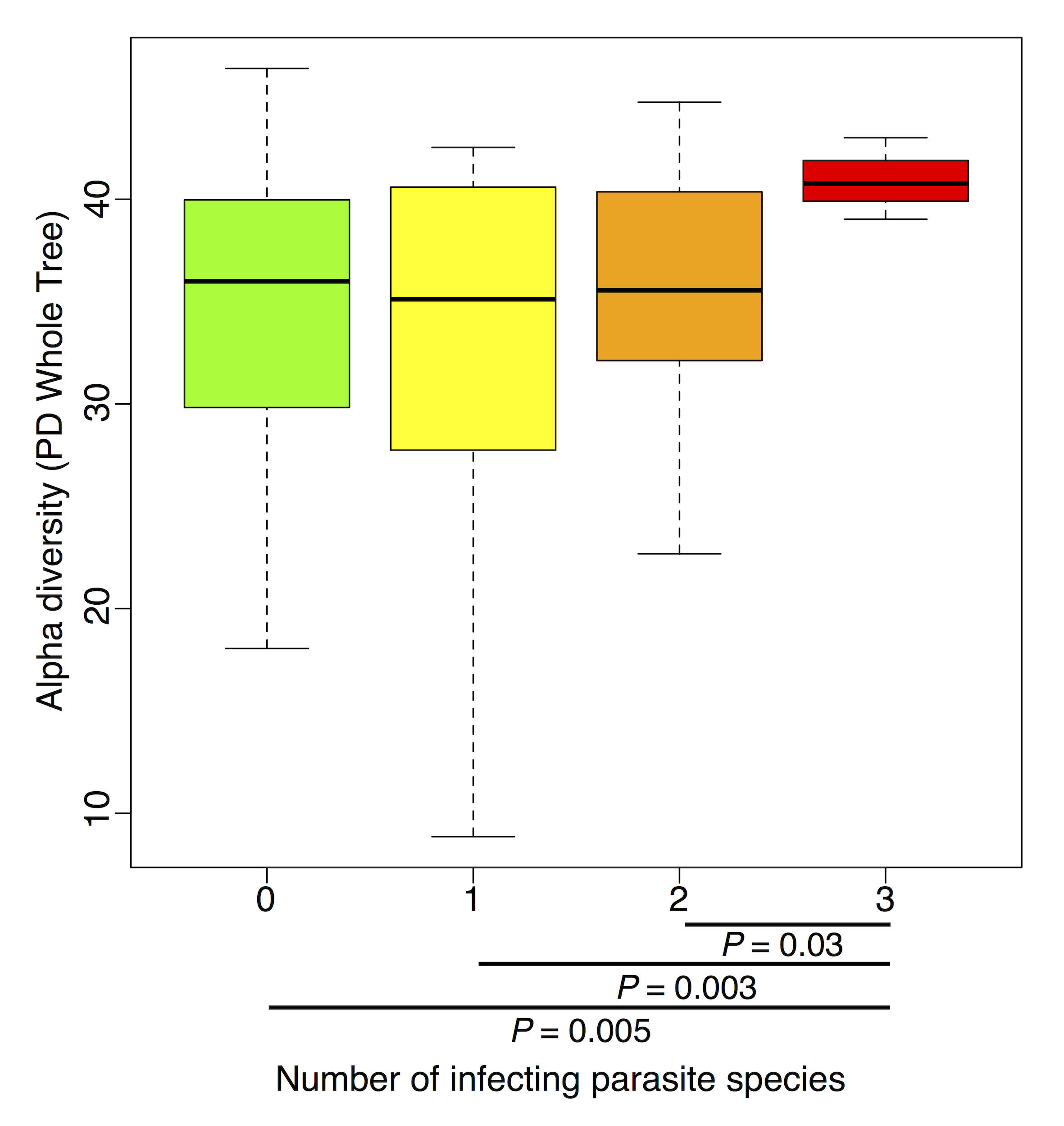

Supplement: S12 Fig — Alpha diversity across individuals grouped by the number of infecting parasite species in the absence of Entamoeba infection (0–3) using the phylogenetic distance whole tree metric. P-values are based on a Welch’s t-test. (TIFF) [file pgen.1005658.s019.tiff]

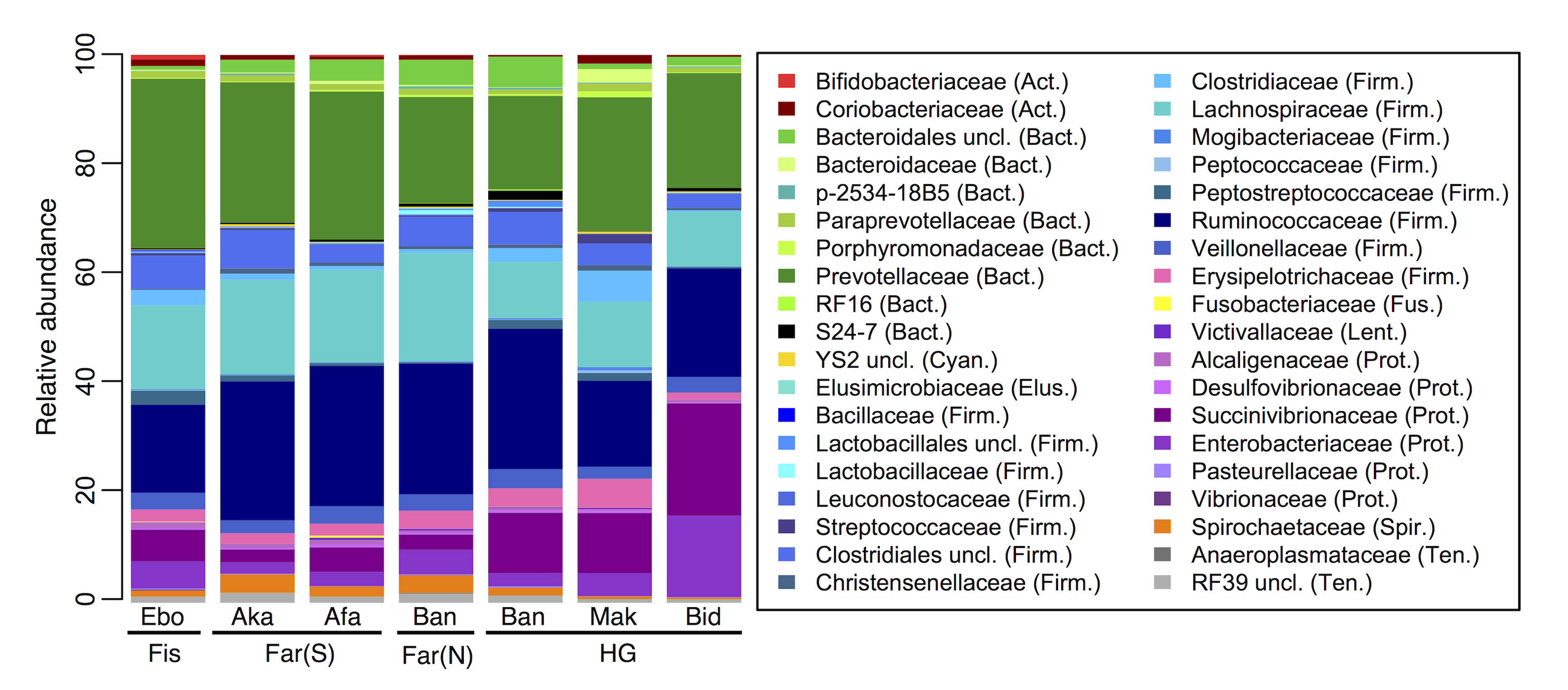

Supplement: S13 Fig — Summary of the relative abundance of taxa (occurring > = 0.1% in at least 4 individuals) for individuals across locations. The Ndtoua village is not considered here as it only includes two individuals. Taxa are colored by phylum (Actinobacteria (Act.) = red, Bacteroidetes (Bact.) = green, Cyanobacteria (Cyan.) = black, Firmicutes (Firm) = blue, Fusobacteria (Fus.) = pink, Lentisphaerae (Lent.) = yellow, Proteobacteria (Prot.) = purple, Spirochaetes (Spir.) = orange, and Tenericutes (Ten.) = gray). Ebo = Ebodie; Aka = Akak; Afa = Afan Essokie; Ban = Bandevouri; Mak = Makoure; Bid = Bidou. Fis = Fishing population; Far(S) = Farmers from the South; Far(N) = Farmers from the North; HG = Hunter-gatherers. (TIFF) [file pgen.1005658.s020.tiff]

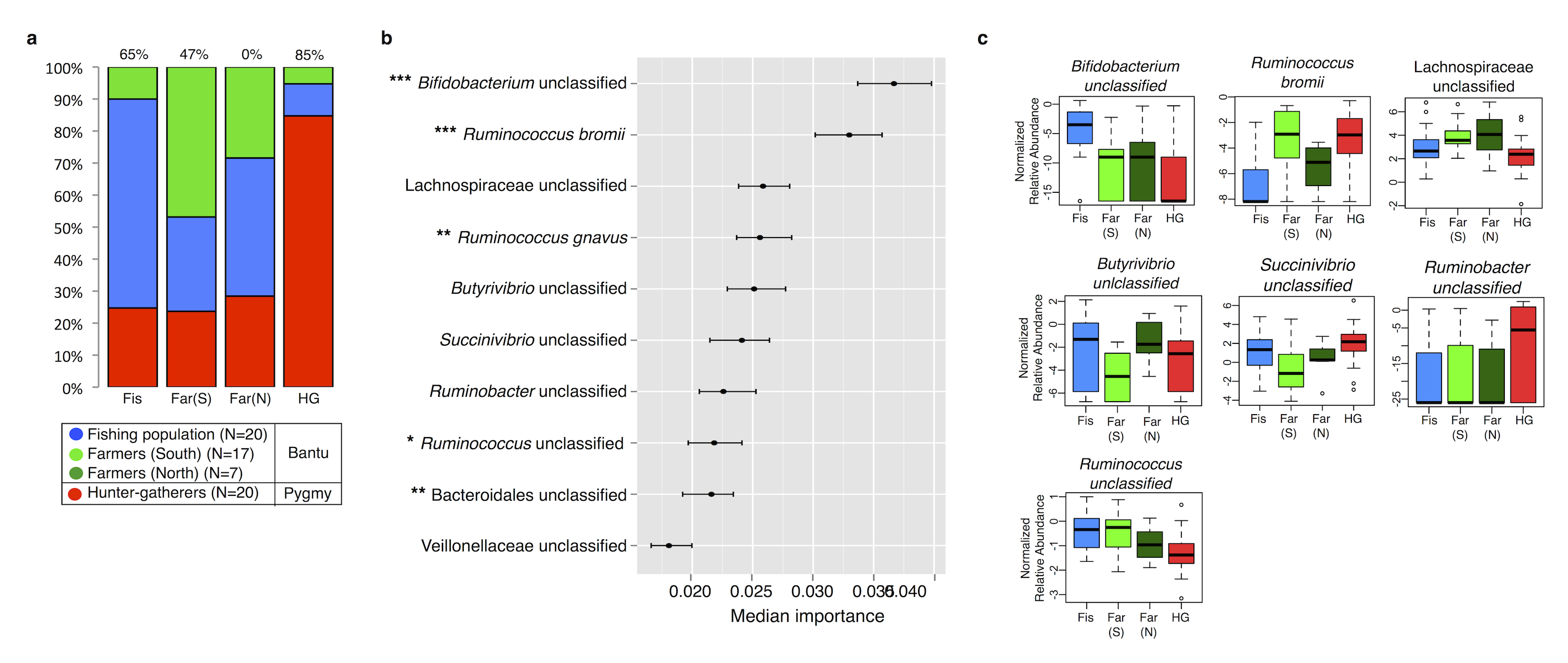

Supplement: S14 Fig — Summary of the ten most discriminating taxa identified by a random forest classifier (RFC) model to be predictive of subsistence group. A RFC with 2000 decision trees was trained on the data (relative abundances of all taxa occurring at > = 0.1% in at least 4 individuals) with 5-fold cross-validation. (a) Bar plot summarizing the predictions of the model for each population. Fis = Fishing population; Far(S) = Farmers from the South; Far(N) = Farmers from the North; HG = Hunter-gatherers. The percent accuracy for each population is indicated above the bars. (b) The ten most important taxa identified in the model ranked by their median importance value. Importance values were calculated as the mean decrease in node impurity. 95% confidence intervals from 1000 random forests are shown. Mean accuracy over the 5 folds was 0.59. P < 0.001, estimated using 100 permutation tests. Taxa that were identified as being significant in an ANOVA are indicated by asterisk(s) (*); q < 0.0001 (***), q < 0.0001 (**), q < 0.001 (*). (c) Normalized relative abundances across subsistence groups of the four taxa (amongst the top ten identified in the RFC model) that are members of the Lachnospiraceae family. q-values are based on an ANOVA. (TIFF) [file pgen.1005658.s021.tiff]

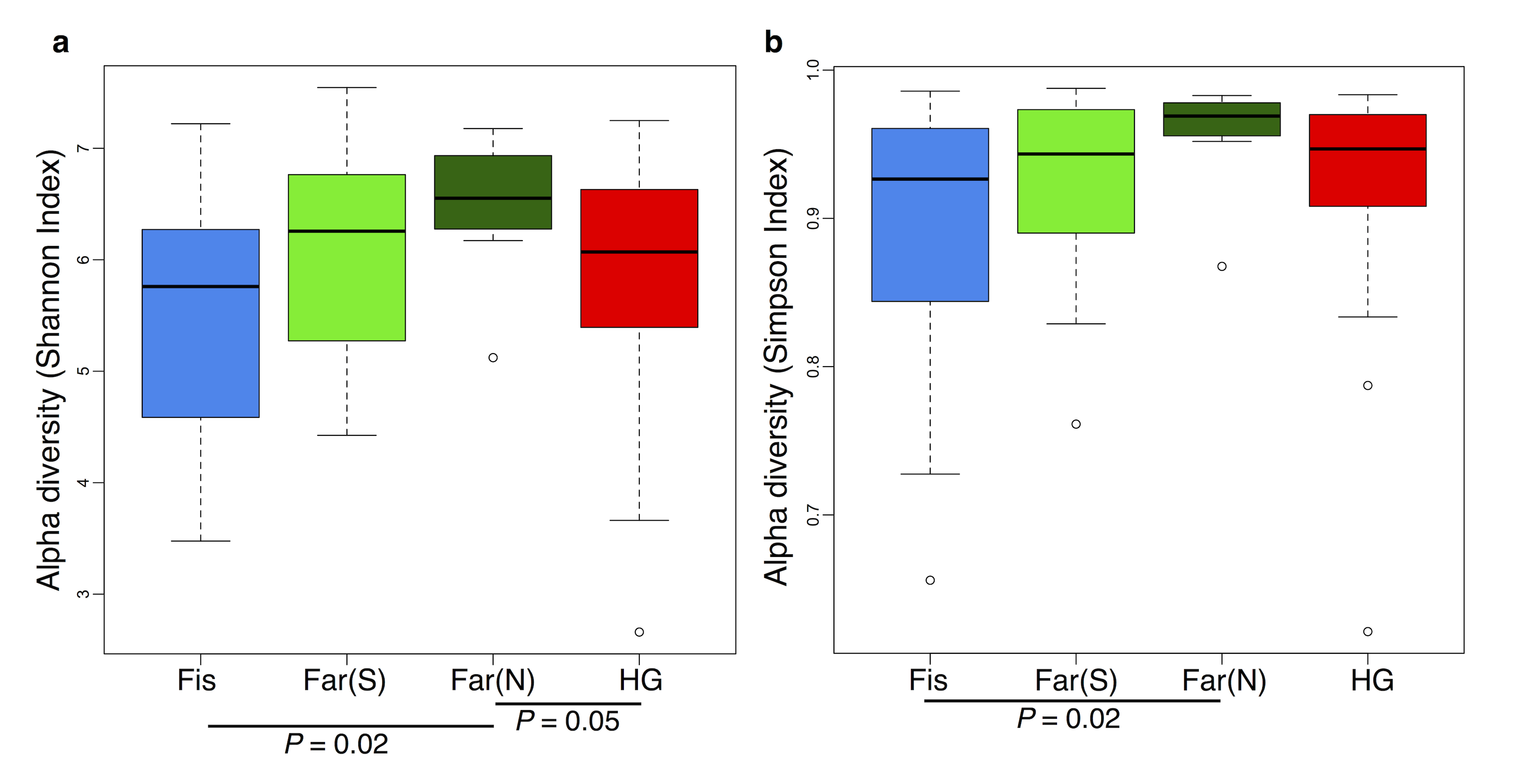

Supplement: S15 Fig — Comparison of individual gut microbial diversity across subsistence modes based on (a) the Shannon Index and (b) the Simpson’s Index metrics for alpha diversity. P-values are based on Welch’s t-tests. Fis = Fishing population; Far(S) = Farmers from the South; Far(N) = Farmers from the North; HG = Hunter-gatherers. (TIFF) [file pgen.1005658.s022.tiff]

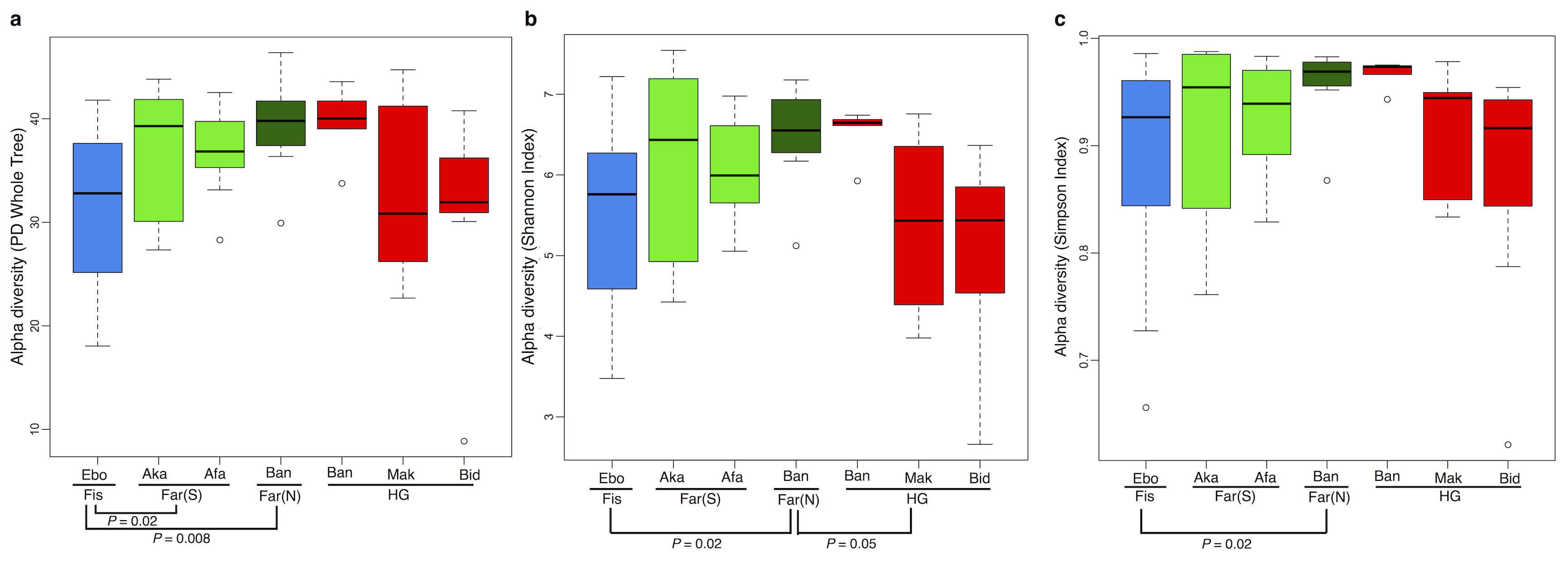

Supplement: S16 Fig — Comparison of individual gut microbial diversity across locations based on (a) the Phylogenetic Distance Whole Tree metric, (b) the Simpson’s Index, and (c) the Simpson’s Index. P-values are based on Welch’s t-tests. There are only 2 individuals from Ndtoua (hunter-gatherers) so they were excluded from analyses by location. Ebo = Ebodie; Aka = Akak; Afa = Afan Essokie; Ban = Bandevouri; Mak = Makoure; Bid = Bidou. Fis = Fishing population; Far(S) = Farmers from the South; Far(N) = Farmers from the North; HG = Hunter-gatherers. (TIFF) [file pgen.1005658.s023.tiff]

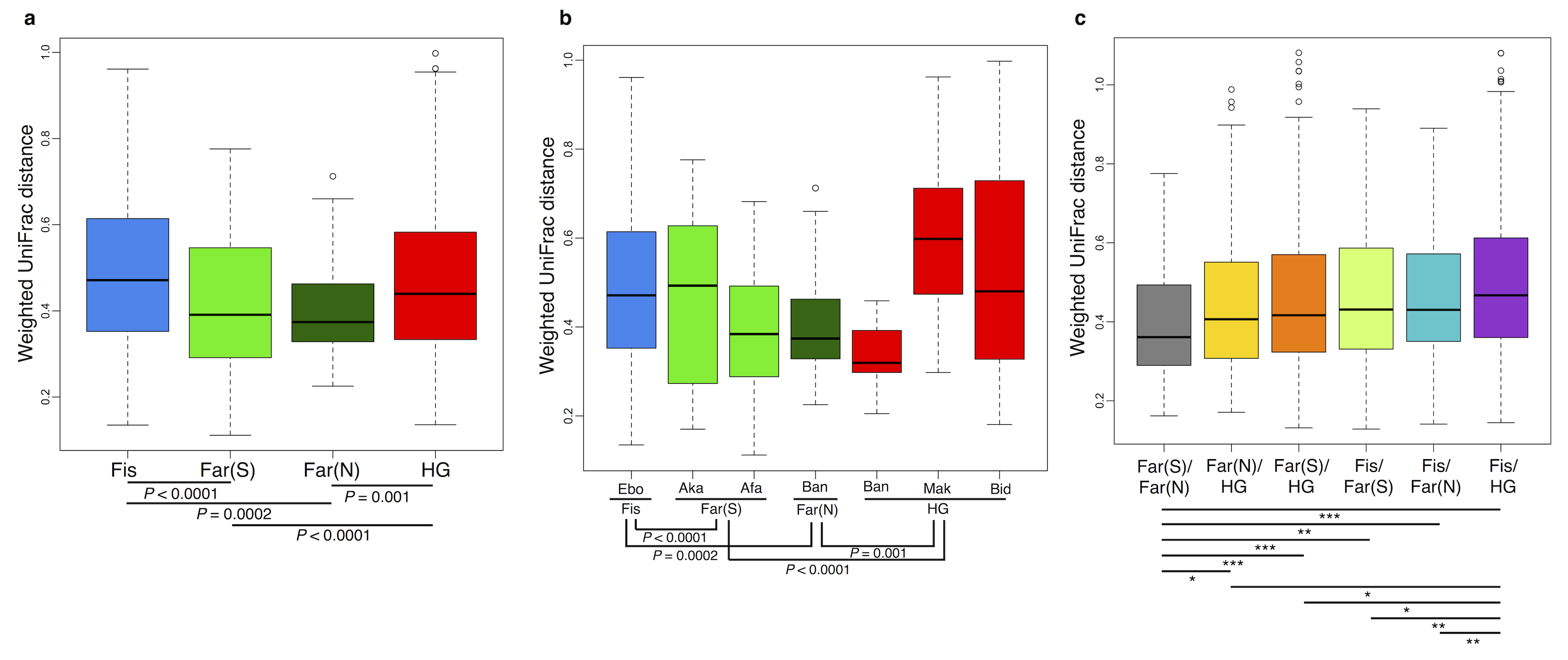

Supplement: S17 Fig — Beta diversity between individuals based on weighted UniFrac distances across (a) subsistence modes and (b) location. (c) Beta diversity between pairs of subsistence groups. All p-values are based on Welch’s t-tests. Subsistence: Fis = Fishing population (blue); Far(S) = Farmers from the South (light green); Far(N) = Farmers from the North (dark green); HG = Hunter-gatherers (red). Locations: Ebo = Ebodie; Aka = Akak; Afa = Afan Essokie; Ban = Bandevouri; Mak = Makoure; Bid = Bidou. There are only 2 individuals from Ndtoua (hunter-gatherers) so they were excluded from analyses by location, but were included in those for subsistence. (TIFF) [file pgen.1005658.s024.tiff]

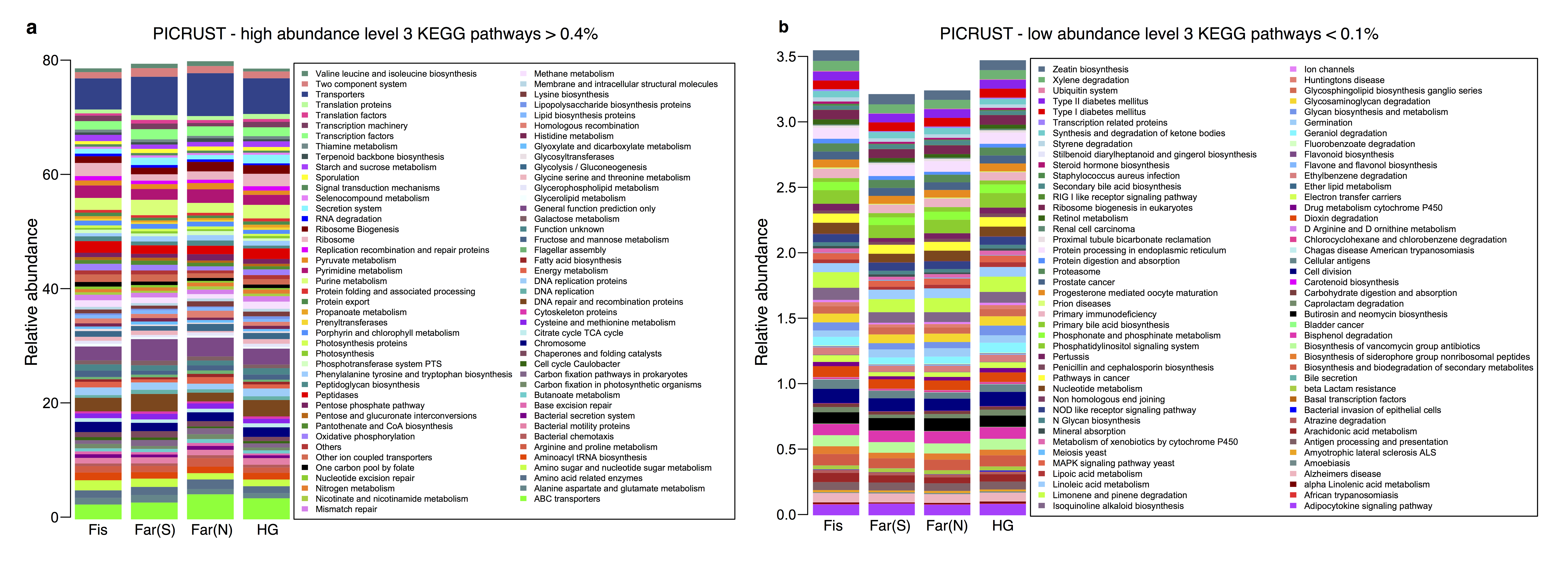

Supplement: S18 Fig — Summary of predicted metagenomic composition for individuals across subsistence modes based on phylotypic investigation of communities by reconstruction of unobserved states (PICRUSt). Relative abundance of (a) the most abundant (>0.4% in at least one group) and (b) least abundant (<0.1% in at least one group) of KEGG (Level 3) pathways. Fis = Fishing population; Far(S) = Farmers from the South; Far(N) = Farmers from the North; HG = Hunter-gatherers. (TIFF) [file pgen.1005658.s025.tiff]
